# Impact of primary care posttraumatic stress disorder (PC-PTSD) on fertility problem of Iranian women with infertility during the COVID-19 pandemic

**DOI:** 10.1186/s12905-024-03102-2

**Published:** 2024-04-27

**Authors:** Mahbobeh Faramarzi, Shiva Shafierizi, Hajar Pasha, Zahra Basirat, Fatemeh Nasiri‑ Amiri, Farzan Kheirkhah

**Affiliations:** 1 Infertility and Reproductive Health Research Center, Health Research Institute, Babol University of Medical Sciences, Babol, Iran; 2 Student Research Committee, Babol University of Medical Sciences, Babol, Iran; 3 Social Determinants of Health Research Center, Health Research Institute, Babol University of Medical Sciences, Babol, Iran

**Keywords:** Infertility, Stress, Pc-PTSD, COVID-19, Ppandemic, Infertility-related stress, Need for parenthood, Relationship concern

## Abstract

**Background:**

Infertility continued to be a major stressor among women with infertility during COVID-19pandemic. This study aimed to evaluate the impact of primary care posttraumatic stress disorder (PC-PTSD) on fertility problem of Iranian women with infertility during COVID-19 pandemic.

**Method:**

In this cross-sectional study, 386 women with infertility completed the questionnaires of PC-PTSD-5 and Fertility Problem Inventory (FPI) at an infertility center between 2020 and 2022.

**Results:**

The mean of fertility problems was 145.20 (± 32.31). In terms of FPI subscales, the means were as follows: Sexual concern 21.80 (± 7.58), social concern 26.53 (± 8.94), relationship concern 26.02 (± 9.18), need for parenthood concern 40.88 (± 8.98), and rejection of childfree lifestyle 29.96 (± 7.69). The highest mean of FPI subscales was related to the need for parenthood concern in women with infertility. The strongest correlation was found between the subscales of sexual concern and social concern followed by sexual concern and relationship concern. The variables of PC-PTSD were a predictor of fertility problems (β = 0.203, *P* < .0001). Additionally, the variables of PC-PTSDwere a predictor of sexual concern (β = 0.248, *P* < .0001), social concern (β = 0.237, *P* < .0001), relationship concern (β = 0.143, *P* < .020), and need for parenthood concern (β = 0.101, *P* < .010). After adjusting for demographic characteristics, there was a significant relationship between FPI with job (β=-0.118, *P* < .031), education (β=-0.130, *P* < .023), living place (β = 0.115, *P* < .035), smoking (β = 0.113, *P* < .036), relationship with husband (β = 0.118, *P* < .027), and PC-PTSD symptom (β = 0.158, *P* < .0001). In addition, the multivariate linear regression showed a significant association between sexual concern and education (β=-0.152, *P* < .008), smoking (β = 0.129, *P* < .018), PC-PTSD symptom (β = 0.207, *P* < .0001); social concern and job (β=-0.119, *P* < .033), PC-PTSD symptom (β = 0.205, *P* < .0001); relationship concern and education (β=-0.121, *P* < .033), living place (β = 0.183, *P* < .001), relationship with husband (β = 0.219, *P* < .0001); and rejection of childfree lifestyle and job (β=-0.154, *P* < .007).

**Conclusion:**

Systematic PTSD screening during COVID-19 pandemic by healthcare providers can be uniquely used to identify, evaluate, and treat trauma-related health conditions in infertility settings, which can link women with infertility to mental health services. This can be novel and useful for future policymakers and practitioners in the infertility field.

## Introduction

Posttraumatic Stress Disorder (PTSD) is a chronic disorder that occurs after exposure to traumatic events [1], when a person experiences severe emotional stress such as war, natural disasters, and life-threatening illnesses [2] such as COVID-19. PTSD is a psychiatric condition related to high levels of functional impairment and poor quality of life [3] Generally, PTSD is diagnosed based on several clusters of symptoms that appear after exposure to extreme stressors [4]. The fifth edition of the Diagnostic and Statistical Manual (DSM-5) pays more attention to behavioral symptoms associated with PTSD and proposes four distinct diagnostic clusters, including re-experiencing, avoidance, negative cognitions and mood, and arousal [1]. PTSD is diagnosed when the DSM-5 symptom criteria algorithm is met and the symptoms cause significant distress and/or impairment. PTSD is now in a new trauma- and stressor-related disorders category, which indicates the cognizance alternation of diseases [5].

The COVID-19 pandemic led to emerging and unprecedented mental health challenges. Millions of people lost their loved ones during the COVID-19 pandemic, which can lead to grief, namely denial, guilt, anger, and PTSD. Trauma exposure and PTSD have been linked with numerous adverse health conditions. PTSD and a peak in the prevalence of mental health problems such as unresolved bereavement and depression occurred during the COVID-19 pandemic [6]. This disease can cause disturbance in individual and family functioning, leading to significant medical, social, financial, and physical health problems [5]. During the COVID-19 pandemic, 25–35% of people suffered from mental stress and anxiety [7, 8]. In particular, with the spread of COVID-19, a number of factors increased people’s stress and anxiety, including the fear of contracting the disease, the spread of false news and rumors, fear of death, prohibitions or restrictions on movement, interference in daily activities, financial and occupational problems, and the reduction of social interactions with colleagues, friends, and family [9, 10].

PTSD can be a risk factor reducing fecundity, increasing the search for infertility treatment and testing among women [11]. Furthermore, infertile persons are at high risk for experiencing trauma and its associated adverse mental health consequences. Infertility as an important problem has a significant negative social impact on infertile couples [12]. It is associated with psychological problems such as anxiety, depression, low self-esteem, sexual distress, stress, and so forth [13]. Women with infertility can experience infertility-related stress that affects their life and infertility treatment approaches [12]. Both infertility and its treatment are associated with stressful experiences and perceived as a stressful and uncontrollable event [14]. Couples with infertility consider it as the most difficult challenge they must overcome. Additionally, women report more anxiety, stress, and depression than men do [15].

Simultaneously, stress-related disorders increase in individuals and societies affected with large disasters such as the global COVID-19 pandemic, especially in women with infertility [16]. According to the literature, COVID-19 affects the reproductive system and response to fertility treatment [17]. Infection with COVID-19 alters the normal immune response with local and systemic injury to organs and tissues. Genital failure can occur after the virus enters the body in both males and females. The possible problem of the male reproductive system is due to rising number of angiotensin-converting enzyme 2 (ACE2) receptors in the testes, Sertoli cells, seminiferous tubule cells, and spermatogonia. Moreover, COVID-19 can also disrupt the function of female reproductive system and affect women’s fertility. The virus may be transmitted sexually and cause infertility or testicular damage [18].

The infertility experience and its treatment are associated with the symptoms of PTSD. A previous study showed that after the appearance of COVID-19, infertility treatment was delayed for several months [12]. This could be a major stressor during COVID-19 pandemic among women with infertility [19].

Nowadays, there is scarce data regarding the impact of COVID-19 pandemic on infertility-related stress. In addition, PTSD demands healthcare service in infertile populations due to the increase in medical complications [5]. The COVID-19 pandemic influenced all areas of life, including infertility and assisted reproductive techniques [20] on the other hand; such psychological problems can affect infertility outcomes. Therefore, it is important to determine infertility-related stress and its related factors in infertile patients before beginning infertility treatment [13]. Although a wide range of psychological consequences can result from trauma exposure, the present study focuses on PC-PTSD symptoms due to their association with poor reproductive health outcomes and the relative infrequency of universal PTSD screening in health infertility settings. Therefore, this research was designed to evaluate the impact of primary care posttraumatic stress disorder on fertility problem of Iranian women with infertility during the COVID-19 pandemic.

## Methods and materials

### Study design and the participants

The present cross-sectional study was performed on women with infertility who enrolled in the two infertility centers in Babol City, Mazandaran Province, Iran, from September 2020 to January 2022 during the COVID-19 pandemic, when the COVID-19 cases were at their peak. The inclusion criteria were as follows: Age ≥ 18 years of age, a minimum of a primary school education, access to the Internet, not currently receiving psychotherapy, not pregnant, and willingness to enter the study, not taking psychiatric drugs in three last months, not report of mental retardation, not self-reporting severe psychiatric disorders, and not substance abuse. The following volunteers were excluded from the study: Incomplete answers to the questionnaires withdrew from further cooperation in research, and experienced stressful life events six months ago.

### Sample size determination

The sample size was determined to be 386 subjects based on pilot data obtained before the study (*p* = .49, α = 0.05, and d = 0.5).

### Data collection

#### Informed consent

was obtained from all subjects after the research project and objectives were explained. Women with infertility who agreed to participate in the study were instructed to complete the questionnaires. Subjects were given the option of completing paper questionnaires inside the clinic or receiving a questionnaire link (via the DigiSurvey® platform) via Telegram® or WhatsApp® to fill out at home, for a week or less. A total of 460 individuals were invited to enter the research, of which 60 were deemed ineligible. A total of 400 women with infertility were enrolled in the study and completed questionnaires. Among the respondents, 151 responded to online questionnaires and 235 responded to paper questionnaires. Fourteen questionnaires were excluded due to inaccuracies in question completion, leaving 386 for data analysis.

### Measurements

#### Fertility problem inventory (FPI)

FPI was used to assess infertility stress and problems. This scale was developed by Newton (1999). The FPI includes 46-item tools and sub-scales, namely sexual concern, social concern, relationship concern, need for parenthood, and rejection of childfree lifestyles. Answers are assigned from 1 (strongly disagree) to 6 (strongly agree). The total score range is from 46 to 276. The higher scores showed higher levels of stress [21]. A cut-off score of FPI scores is considered by, a raw score of 167 or above as assessed in females and of 147 or above as assessed in males [22]. The validity and reliability of the Persian version were evaluated by Samani et al. (2017). The overall integrity was 0.87. Cronbach’s alpha for all sub-scales was more than 0.7 [23].

### Primary care posttraumatic stress disorder (PC‑PTSD‑5)

This scale evaluates posttraumatic stress symptoms, which is a five-question self-report screening. Scores are ranged on a binary scale of 0 to 1 (0 = no; 1 = yes). The total score consists of adding the score of five questions. A cut-off score of 3 is considered for PTSD symptoms [5]. Higher scores show increased posttraumatic stress symptoms. The Cronbach’s alpha for the Iranian version of PC-PTSD was 0.7. In this study, a validated Persian version was done [24].

### Statistical analysis

We used the mean and standard deviation for describing infertile women’s characteristics. Simple and multivariate linear regression models analyzed the predictors for fertility problem symptoms and their subscales. Age, Husband age, job, education level, living place, smoking, relationship with husband, history of assisted reproductive technology failure, pc-PTSD score as independent variables, fertility problem symptoms, and its subscales score as dependent variables were considered. For data analysis, IBM SPSS Statistics Version 22 was used. P-value < 0.05 was the significance level.

## Results

The age range of most women with infertility was 25–35 years (58.5%) and their husbands were aged 30–40 years (67.5%). Most women with infertility held high school diploma (50.4%), lived in rural areas (58.2%), were housewives (74.25%), and their husbands were self-employed (63.2%). The majority of women with infertility were satisfied with their husbands (96.7%). The infertility duration in the majority of participants was less than 10 years (82.6%). The mean duration of Marriage (years) was 7.6 ± 4.68. The demographic characteristics of the research population are summarized in Table 1.

**Table 1:** Descriptive results for demographics characteristics, and FPI in women with infertility (*n* = 386)

Variable	Frequency (n)	Percent (%)	Variable	Frequency (n)	Percent (%)
**Age(yr)**			**Education**		
< 25	41	11.1	≤Diploma	177	50.4
25–35	216	58.5	University	174	49.6
> 35	112	30.4	**Relationship with husband**		
**Husband age(yr)**			satisfied	353	96.7
< 30	50	13.3	unsatisfied	12	3.3
30–40	253	67.5	**Living place**		
**> 40**	72	19.2	rural	223	58.2
**Infertility time(yr)**			urban	160	41.8
≤ 10(yr)	280	82.6	**ART failure**		
> 10(yr)	59	17.4	no	185	48.6
**Job**			yes	196	51.4
Housewives	285	74.3	**Variable**	**Mean(SD)**	**Min(Max)**
Employee	99	25.7	**FPI**	145.20(32.31)	(69–223)
**Husband job**			Sexual concern	21.80(7.58)	(8–43)
Unemployed	3	0.8	Social concern	26.53(8.94)	(10–52)
worker	85	22.4	Relationship concern	26.02(9.18)	(10–56)
Employee	52	13.6	Need for parenthood concern	40.88(8.98)	(13–60)
Sef-employed	241	63.2	Rejection of childfree lifestyle	29.96(7.69)	(12–48)

*Note* Range of scores: FPI Fertility problem inventory: 46–276, Sexual concern: 8–48, Social concern: 10–60, Relationship concern: 10–60, Need for parenthood: 10–60, Rejection of childfree lifestyle: 8–48

The mean of fertility problems was 145.20 (± 32.31). In terms of fertility problem subscales, the means were as follows: Sexual concern 21.80 (± 7.58), social concern 26.53(± 8.94), relationship concern 26.02(± 9.18), need for parenthood concern 40.88(± 8.98), and rejection of childfree lifestyle 29.96(± 7.69). The highest mean of FPI domains was related to the need for parenthood concern in women with infertility. There was a significant correlation between the domains of fertility problems (*P* < .0001). The strongest correlation was observed between the domains of sexual concern and social concern, followed by sexual concern and relationship concern. (Table 2).

**Table 2:** Correlation coefficient matrix between pc-PTSD, FPI, and its dimensions in women with infertility (*n* = 386)

	Pc-PTSD	FPI	SC	SOC	RC	NPC	RCL
**Pc-PTSD**	1						
**FPI**	0.203 0.000	1					
**SC**	0.248 0.000	0.756 0.000	1				
**SOC**	0.237 0.000	0.804 0.000	0.577 0.000	1			
**RC**	0.143 0.005	0.779 0.000	0.567 0.000	0.557 0.000	1		
**NPC**	0.101 0.048	0.762 0.000	0.438 0.000	484 0.000	0.401 0.000	1	
**RCL**	0.046 0.369	0.702 0.000	0.330 0.000	0.418 0.000	0.404 0.000	0.561 0.000	1

*Note* Statistical significance was determined by calculating Pearson’s correlational analysis

**SC**: Sexual concern, **SOC**: Social concern, **RC**: Relationship concern; **NPC**: Need. For parenthood concern, **RCL**: Rejection of childfree lifestyle

There was a significant association between PC-PTSD with all the domains of FPI except for the rejection of childfree lifestyle items. The results of this study on predictors of fertility problems on concerning PC-PTSD of women with infertility revealed a significant positive association between fertility problems and PC-PTSD. Based on the results of linear regression analysis, the variable of PC-PTSD (β = 0.203, *P* < .0001) was a predictor of fertility problems. PC-PTSD factors explained 4.1% of fertility problem variance. Furthermore, there was a significant positive association between fertility problem subscales and PC-PTSD. According to linear regression analysis, the variables of PC-PTSD were a predictor of sexual concern (β = 0.248, *P* < .0001), social concern (β = 0.237, *P* < .0001), relationship concern (β = 0.143, *P* < .020), and need for parenthood concern (β = 0.101, *P* < .010). In other words, more PC-PTSD symptom is associated with more frequent fertility problems and their subcomponents except for the rejection of a childfree lifestyle in women in infertility. The regression results revealed that 6.1% and 5.6% of sexual and social concerns in women with infertility could be explained based on Pc-PTSD symptoms, respectively (Table 3).

**Table 3:** Results of linear regression model for Pc-PTSD with FPI in women with infertility (*n* = 386)

Model	Dependent Variable	Independent Variable	B	SE	Beta	t	*R* ^2^	*P* Value	CI%	
									Low	High
1	FPI	Pc-PTSD	5.393	1.325	0.203	4.069	0.041	0.0001	2.787	7.998
2	**SC**	Pc-PTSD	1.542	0.308	0.248	5.013	0.061	0.0001	0.937	2.147
3	SOC	Pc-PTSD	1.740	0.364	0.237	4.784	0.056	0.0001	1.025	2.455
4	**RC**	Pc-PTSD	1.078	0.381	0.143	2.832	0.020	0.005	0.330	1.827
5	**NPC**	Pc-PTSD	0.743	0.374	0.101	1.984	0.010	0.048	0.007	1.479
6	**RCL**	Pc-PTSD	0.290	0.322	0.046	0.900	0.002	0.369	− 0.343	0.922

*Note***SC**: Sexual concern, **SOC**: Social concern, **RC**: Relationship concern; **NPC**: Need. For parenthood concern, **RCL**: Rejection of childfree lifestyle

After adjusting the variables of demographic characteristics by applying multiple linear regression, there was significant relationship between fertility problem and job (β=-0.118, *P* < .031), education (β=-0.130, *P* < .023), living place (β = 0.115, *P* < .035), smoking (β = 0.113, *P* < .036), relationship with husband (β = 0.118, *P* < .027), and PC-PTSD symptom (β = 0.158, *P* < .0001). Moreover, the multivariate linear regression showed a significant association between sexual concern and education (β=-0.152, *P* < .008), smoking(β = 0.129, *P* < .018), PC-PTSD symptom (β = 0.207, *P* < .0001); social concern and job (β=-0.119, *P* < .033), PC-PTSD symptom (β = 0.205, *P* < .0001); relationship concern and education (β=-0.121, *P* < .033), living place (β = 0.183, *P* < .001), relationship with husband (β = 0.219, *P* < .0001); and rejection of childfree lifestyle and job (β=-0.154, *P* < .007)(Table 4).

**Table 4:** Results of multiple linear regression model for Pc-PTSD with FPI^*^ subscales in women with infertility (*n* = 386)

Variables	Sexual concern (*R*^2^ = 0.121)				Social concern (*R*^2^ = 0.105)				Relationship concern (*R*^2^ = 0.133)				Need. for parenthood concern (*R*^2^ = 0.055)				Rejection of childfree lifestyle (*R*^2^ = 0.064)			
	Beta	*P*	CI%		Beta	*P*	CI%		Beta	*P*	CI%		Beta	*P*	CI%		Beta	*P*	CI%	
			L	H			L	H			L	H			L	H			L	H
**Age**	**0.014**	**0.792**	**− 0.11**	**0.15**	**− 0.054**	**0.316**	**− 0.24**	**0.08**	**− 0.009**	**0.868**	**− 0.17**	**0.15**	**− 0.074**	**0.186**	**− 0.27**	**0.05**	**− 0.098**	**0.078**	**− 0.26**	**0.01**
**Job**																				
Housewives (Ref)																				
Employee	**− 0.044**	**0.430**	**-2.55**	**1.09**	**− 0.119**	**0.033**	**-4.57**	**− 0.19**	**− 0.056**	**0.309**	**-3.33**	**1.06**	**− 0.076**	**0.183**	**-3.78**	**0.72**	**− 0.154**	**0.007**	**-4.46**	**− 0.71**
**Education**																				
≤Diploma(Ref)																				
University	**− 0.152**	**0.008**	**-3.92**	**− 0.59**	**− 0.069**	**0.234**	**-3.21**	**0.79**	**− 0.121**	**0.033**	**-4.19**	**− 0.17**	**− 0.099**	**0.096**	**-3.81**	**0.32**	**− 0.048**	**0.416**	**-2.43**	**1.01**
**Living place**																				
Rural(Ref)																				
Urban	**0.075**	**0.172**	**− 0.49**	**2.75**	**0.086**	**0.125**	**− 0.42**	**3.47**	**0.183**	**0.001**	**1.36**	**5.27**	**0.023**	**0.693**	**-1.60**	**2.41**	**0.061**	**0.285**	**− 0.76**	**2.58**
**Smoking**																				
**No**(Ref)																				
**Yes**	**0.129**	**0.018**	**0.59**	**6.16**	**0.070**	**0.200**	**-1.16**	**5.52**	**0.085**	**0.114**	**− 0.66**	**6.06**	**0.104**	**0.065**	**− 0.21**	**6.69**	**0.036**	**0.518**	**-1.93**	**3.82**
**relationship with husband**																				
Satisfied(Ref)																				
Unsatisfied	**0.063**	**0.240**	**-1.72**	**6.85**	**0.088**	**0.106**	**− 0.90**	**9.38**	**0.219**	**0.0001**	**5.63**	**15.96**	**− 0.031**	**0.573**	**-6.82**	**3.78**	**0.101**	**0.068**	**− 0.31**	**8.53**
**Pc-PTSD**																				
No(Ref)																				
Yes	**0.207**	**0.001**	**0.62**	**1.93**	**0.205**	**0.0001**	**0.72**	**2.28**	**0.089**	**0.096**	**− 0.12**	**1.46**	**0.077**	**0.170**	.**-24**	**1.37**	**0.017**	**0.763**	**− 0.57**	**0.78**
**Constant**		**0.0001**	**7.99**	**24.86**		**0.0001**	**13.32**	**33.54**		**0.025**	**1.46**	**21.78**		**0.0001**	**35.49**	**56.35**		**0.0001**	**23.03**	**40.42**

*Multiple linear regression showed a significant relationship between FPI with all of the noted variables except age (*p* < .05), R^2^ = 0.135

## Discussion

This research focused on the impact of primary care post-traumatic stress disorder on fertility problem of Iranian women with infertility from September 2020 to January 2022 during the COVID-19 pandemic.

The present study indicated that PTSD and fertility problems are associated with each other. A history of PTSD can increase infertility-related stress. In agreement with our study, Roozitalab et al. (2021) demonstrated a direct significant relationship between posttraumatic stress disorder and level of stress in women with infertility [25]. While Azad et al. (2022) reported that COVID-19 pandemic did not affect infertility-related stress in women with infertility [12]. Nearly half of the women with infertility met the criteria for any psychological distress. Fertility status is an enduring condition that has a significant association with mental health outcomes. Many covariates make significant independent contributions to psychological distress. The COVID‑19 pandemic hurts the quality of life and the mental health of women seeking fertility services [26]. Furthermore, the review of literature highlighted that the couples with infertility also confronted the psychological effect of COVID‑19 pandemic due to quarantine, social distancing, travel restrictions, and cancellation of treatment [27]. Therefore, appropriate and timely psychological counseling along with organizational and social support are essential for coping with negative impacts of COVID‑19 pandemic on women with infertility [26].

The findings of our study after adjusting for other variables indicated that PC-PTSD was a significant risk factor for sexual concern subscales of fertility problems among women with infertility. The participants presenting with more PC-PTSD symptoms had higher sexual concerns than those who had fewer symptoms of PC-PTSD. A previous study revealed that women reported higher stress levels in subscales of sexual concern and need for parenthood [28]. Pasha (2020) reported that one of the important concerns of couples is sexual relationships during a pandemic and that COVID-19 gives rise to public health concerns over sexual health [29]. A review of literature showed that infertile women were unsatisfied with marriage as their ultimate dream of marriage was to give birth to children. Their dissatisfaction also had a direct effect on sexual life and they had a reduced desire to have sex. Although procreation is the driving force for sexual intercourse among couples, consistent failure of attempts to have children can decrease sexual activity with husbands [30].

The gathered data revealed that PC-PTSD was a significant risk factor for the social concern subscale of fertility problems. In other words, the subjects with more PC-PTSD symptoms had higher social concerns than those who had fewer symptoms of PC-PTSD, which was in line with the study of Awtani et al. (2017) [28]. Additionally, Arba˘g et al. (2023) reported a decrease in social relations among women with infertility due to the COVID-19 pandemic [20]. Studies revealed that social support protected people against the adverse health effects of accepting infertility during the COVID-19 pandemic and that more social support was related to less emotional distress [31, 32].

It seems that women with infertility used to be exposed to uncomfortable questions raised by their friends and relatives even before COVID-19. This pandemic prompted them to refuse contact or hide from others and socially isolat themselves [33]. A review of literature revealed that women with infertility experienced social isolation by staying at home, avoiding meeting their friends, and not speaking about receiving infertility treatment [34]. The decrease in social relationships during COVID-19 pandemic can reduce not only exposure to questions about infertility and social gatherings one attends but also decrease the social gatherings where they feel comfortable with each other. Also, infertile women stated that the inactivity of infertility clinics during the pandemic had a negative effect on their lives. They experienced sadness, hopelessness, exhaustion, unreliability, and anger from waiting [20], which increased infertility stress. Discontinuation of fertility care during COVID-19 pandemic had a significant psychological impact on women with an increase in stress and anxiety [35]. In this regard, Tokgoz et al. (2022) indicated that the level of fear and anxiety was found to be higher in women with infertility whose ART cycles were postponed due to COVID-19 outbreak [36]. There was higher anxiety and distress among women with infertility with postponed or interrupted treatment strategies during the COVID-19 pandemic. The COVID-19 pandemic and delayed infertility treatment was important stressors among women with infertility [37]. Therefore, psychological intervention and collecting accurate information about resolving sexual and social concerns should be attempted, especially during this difficult period [35].

The finding of the present study showed that participants with unsatisfied relationships with their spouses had higher stress levels associated with fertility problems as well as relationship concerns domain compared to subjects whose were satisfied relationships with their husbands. The review of literature showed that infertility can increase marital conflicts and problems, which is associated with serious consequences for social well-being. Poor marital quality can be linked with many family and community problems. Infertility is considered a social concern decreasing the emotional bonds of couples and deteriorating the quality of married life [38, 39]. This can be difficult as the marital relationship is considered the most important factor of support in relation with infertility treatment strategy. This finding highlights the need for early detection of marital conflicts in couples with infertility and marriage enrichment intervention, which creates positive changes as the couple practices healthy interaction skills.

The results of the present study revealed that higher stress levels in women with infertility were in the domains of need for parenthood followed by rejection of a childfree lifestyle, which was in line with the findings of Shayesteh-Parto et al. (2023) study [38]. In its explanation, it can be stated that in underdeveloped countries, people without children are often considered to not have children as those who voluntarily select not to have children, while in developed societies, childfree is voluntary and is an appropriate option for growth and development [12]. For most women, giving birth is an important part of their life, and all societies place great value on having children [40]. Tabong et al. (2013) reported that couples with infertility suffer from social effects of childlessness. Couples without children are socially stigmatized and denied leadership roles in their communities. Both men and women with infertility are deprived of membership in the ancestral world, thereby losing the opportunity to live again. It seems that health policymakers should be more concerned about infertility and provide optimal solutions in this field [30].

The finding of this study also demonstrated that the fertility problem was significantly associated with some socio-demographic data, such as job, educational status, and living place. The infertility problem, social concern, and rejection of a childfree lifestyle were lower in employed women than in unemployed women. In contrast with our study, Azad et al. (2023) showed that total infertility stress was significantly associated with employment [12]. In the interpretation of this finding, it can be acknowledged that employed women experience less stress due to their educational background, financial position, professional career, and high socioeconomic status, which can lead to a more powerful position in their community. In contrast, homemakers from a lower socio-economic background are more likely to be challenged with the social stigma of infertility. In a similar investigation, Esra Arba˘ et al. (2023) stated that the fertility treatment protocol affects women with infertility not only psychologically and physiologically but also economically. The women with infertility stated that the costs of treatment were superimposed on their pandemic-related financial difficulty [20]. It seems that unemployed women suffer more from these difficult financial conditions, which can exacerbate their stress.

Our study also indicated a significant negative relationship between educational status level of subjects with two FPI subscales of sexual concerns and relationship concerns. Participants with a university education had fewer fertility problems, sexual concerns, and relationship concerns than those with a high school diploma who experienced higher fertility problems. A review of literature showed that total infertility stress was significantly associated with educational status. Educational levels of subjects revealed a significant adverse correlation with some FPI subscales [12]. In another study, there was a significant association between FPI scores and educational status in couples with infertility [41], While Teklemicheal et al. (2022) revealed that fertility problems are not related to educational status [42].

According to demographic characteristics of participants, higher stress levels of fertility problems and the relationship concerns domain were observed among women with infertility that lived in urban areas than those who lived in rural parts. In agreement with the present study, Dierickx et al. (2018) revealed a strong social pressure on women living in cities with infertility [43]. Besides, Kucuk et al. (2022) stated that cultural and socio-demographic features influence the level of stigmatization of women with infertility; the higher the level of stigma, the more difficult for women with infertility to cope with infertility stress [44].

Current data indicate that women with infertility who smoked had more infertility-related stress as well as sexual concerns domains than those who were non- smokers. Stress can be considered a significant risk factor for cigarette smokers [45]. A review of literature has reported that women are prone to increased stress due to infertility [46]. Drug addiction and alcohol are prevalent in individuals with infertility [47]. Moreover, PTSD is associated with increased health-compromising behaviors such as tobacco use [5]. There are several opinions on the impact of stress and smoking behaviors. Smokers often use cigarettes to alleviate stress [45]. However, previous studies have reported that while smoking may provisionally decrease stress, it may give rise to or worsen negative emotional conditions and increase negative coping strategies leading to overall higher stress levels in individuals [48]. Therefore, more attention on the part of health policymakers is essential for healthy or unhealthy coping strategies in populations with infertility.

## Conclusion

It is suggested to use screening approaches for mental health that help healthcare professionals determine potential stressors for fertility problems during COVID-19 pandemic, which can improve fertility problems. Identifying women with infertility, who have active PTSD symptoms related with the infertility treatment due to high rates of undetected PTSD and low rates of mental health services is especially important.

## Data Availability

The data supported during the present study are available from the corresponding author upon reasonable request.
